# Nanosensors and Microsensors for Body Fluid Monitoring: Various Analyte Detection and Construction Solutions

**DOI:** 10.3390/ijms26115001

**Published:** 2025-05-22

**Authors:** Nikola Lenar, Beata Paczosa-Bator

**Affiliations:** Faculty of Materials Science and Ceramics, AGH University of Krakow, Mickiewicza 30, PL-30059 Krakow, Poland

**Keywords:** body fluids monitoring, nanosensors, microsensors, wearable, implantable sensors, lab-on-a-chip systems, microfluidic sensors, MEMS

## Abstract

This review provides a comprehensive overview of the recent advancements in nanosensors and microsensors for body fluid monitoring. The principles behind sensor technologies, their applications in healthcare, and the types of body fluids that they analyze are described in the scope of this paper. Additionally, this review discusses emerging trends, challenges, and future perspectives in this field. The first two sections explore various body fluids and their diagnostic significance and discuss the fundamentals and classification of nanosensors and microsensors. The main aim of this paper is to highlight recent advancements in nanosensors for body fluid monitoring and to examine the role of microsensors in healthcare diagnostics. Innovative solutions such as microfluidic-based sensors, lab-on-a-chip systems, MEMS-based sensors, and wearable and implantable sensors are discussed in this section. Various construction solutions for microsensors and nanosensors have also been compiled and compared based on their target analytes, which are widely present in body fluids. The following sections review technologies and trends, including AI integration and flexible sensors, and discuss challenges and future perspectives in the development and application of sensors. The conclusion includes a summary of key findings and the future outlook for nanosensors and microsensors in personalized medicine.

## 1. Introduction

Sensors have become essential to modern healthcare, particularly with the development of microsensors and nanosensors that are designed for the detection and quantification of various biological, chemical, and physical parameters of body fluids. These sensors are leading the way in medical diagnostics, enabling early disease detection, the continuous monitoring of biochemical markers, and the management of chronic conditions. The development of biosensors has revolutionized our ability to detect electrolytes and other analytes such as glucose, lactate, and disease biomarkers with high sensitivity and specificity [1,2]. Recent advancements in wearable sensors and implantable devices have enabled the continuous monitoring of vital signs and biochemical markers. For instance, microfluidic biosensors that are integrated into wearables can measure the wearer’s sweat loss, sweating rate, and sodium concentration in real-time, offering valuable insights into their hydration status and electrolyte balance [3,4]. Furthermore, the integration of microsensors and nanosensors with the internet of things (IoT) enables real-time remote patient monitoring. This allows healthcare providers to continuously track patients’ health data, respond promptly to changes, and support better patient involvement in and adherence to treatment plans [5,6]. These advancements contribute to better health outcomes and personalized medicine.

Nanosensors and microsensors offer significant advantages over traditional sensing technologies, particularly in the context of body fluid monitoring. Their miniaturized structure provides enhanced capabilities that make them highly suitable for real-time diagnostic applications. First and foremost, nano- and microsensors exhibit high sensitivity and selectivity due to their small size [7]. This allows for a higher surface area/volume ratio, allowing for the detection of analytes at low concentrations with high specificity. This feature is essential for early disease detection, where biomarkers such as glucose and lactate may be present in trace amounts in body fluids [8]. Moreover, thanks to their size, nanosensors and microsensors can be integrated into wearable devices that monitor body fluids such as sweat or interstitial fluid, offering a non-invasive (or minimally invasive) alternative to traditional blood tests. This reduces patient discomfort and promotes continuous monitoring without the need for invasive procedures [9]. The miniaturization of these sensors results in faster response times, enabling real-time monitoring and immediate feedback, which is crucial in acute care settings where timely intervention can be lifesaving. For example, wearable sensors for continuous glucose monitoring provide constant updates to guide insulin therapy [10]. Nanosensors and microsensors can be engineered to detect multiple analytes simultaneously, allowing doctors to obtain a comprehensive view of a patient’s health status from a single fluid sample. This multiplexing ability significantly improves the efficiency of diagnostics and reduces the need for multiple tests.

The implementation of microsensors and nanosensors allows for the real-time monitoring of body fluids. Real-time monitoring is revolutionizing modern healthcare, allowing for early disease detection, personalized treatment strategies, and continuous health management [11,12]. Body fluids such as blood, saliva, sweat, urine, tears, and interstitial fluid contain critical biomarkers that reflect an individual’s physiological and pathological state [13]. Traditional diagnostic methods rely on centralized laboratory analyses, which can lead to delays, increased healthcare costs, and limited accessibility. In contrast, real-time biosensing technologies facilitate rapid clinical decision-making, improving patient outcomes and reducing hospital visits. The integration of real-time monitoring into daily life is transforming personalized healthcare, empowering individuals to take proactive measures to manage their health. Technologies such as continuous glucose monitoring (CGM) systems have revolutionized diabetes care, providing instant glucose readings to help patients adjust their insulin doses in real time [14,15,16,17]. Similarly, monitoring the electrolytes in an individual’s sweat can prevent dehydration and heat-related illnesses, particularly in athletes, military personnel, and outdoor workers [18]. The continued advancement of microsensors and nanosensors is paving the way for accessible, continuous, and minimally invasive health monitoring, which will ultimately improve health outcomes and users’ quality of life.

**Figure 1:**
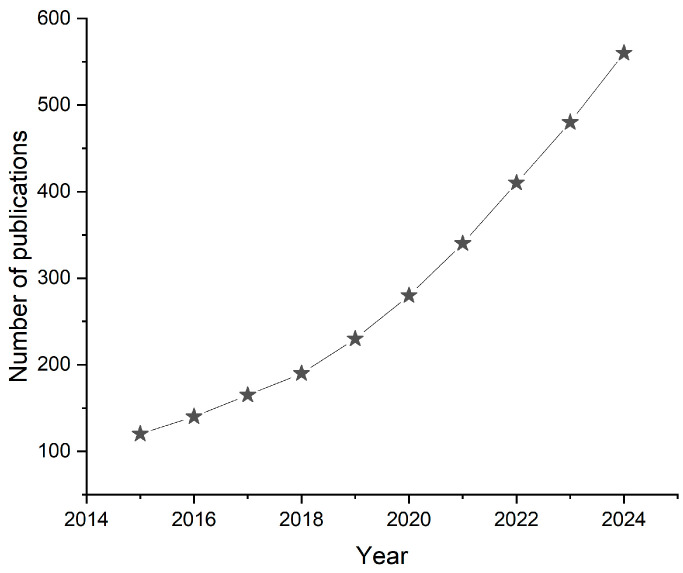
Annual number of publications, from 2015 to 2024, related to miniaturized sensors for body fluid monitoring. Data were obtained from a Scopus search using keywords: “miniaturized sensor”, “body fluid”, “biosensor”, “wearable”, “implantable”, and “lab-on-a-chip”.

In recent years, there has been a notable increase in research focused on miniaturized sensors for body fluid monitoring. This surge is evident in the rising number of publications on the topic, which is depicted in Figure 1. This upward trend underscores the scientific community’s growing interest in developing compact, efficient, and non-invasive diagnostic tools. Factors that contribute to this growth include advancements in miniaturization, an increased demand for point-of-care diagnostics, and the integration of sensors with wearable and implantable devices.

This review is a comprehensive overview of the recent advancements in nanosensors and microsensors for body fluid monitoring. It explores the types of body fluids that are analyzed, the principles behind sensor technologies, and the application of these technologies in healthcare. Additionally, this review discusses emerging trends, challenges, and future perspectives in this rapidly evolving field.

## 2. Types of Body Fluids and Their Diagnostic Importance

Body fluids are vital biological liquids that facilitate numerous physiological functions, including nutrient transport, waste removal, and the maintenance of homeostasis. The body’s fluids are primarily composed of water, but also contain many other substances. One such group of substances is electrolytes, which includes sodium, potassium, magnesium, phosphate, and chloride. Another group includes metabolites such as oxygen, carbon dioxide, glucose, lactate, and urea. A third important group of substances, proteins, most of which are vital for our existence, is contained within our body’s water. Examples of proteins include coagulation factors, immunoglobulins, albumin, and various hormones [13]. Examples of body fluids are blood, saliva, sweat, urine, tears, and interstitial fluid, each of which offers unique insights into an individual’s health status.

Blood is a complex fluid composed of plasma, red and white blood cells, and platelets. It serves as the primary medium for transporting oxygen, nutrients, and metabolic waste products. Blood contains glucose, cholesterol, proteins, disease biomarkers for heart disease such as cardiac troponins, and cancer markers like prostate-specific antigen (PSA) [19]. Blood analysis is fundamental in medical diagnostics, enabling the detection of various biomarkers that are indicative of health and disease states. Real-time blood monitoring is essential for managing diabetes, cardiovascular diseases, and infectious conditions.

Saliva is a mixture of oral fluids, including secretions from the salivary glands, cellular material, and food particles. Saliva contains hormones (cortisol), enzymes (amylase), and pathogens (bacteria, viruses), making it valuable for stress monitoring, infectious disease detection, and detecting hormonal imbalances [20,21]. Saliva-based diagnostics have been widely explored in COVID-19 testing and oral disease detection.

Sweat is an exocrine secretion that is primarily involved in thermoregulation. It contains electrolytes such as sodium and chloride, and its analysis can offer insights into an individual’s hydration status and electrolyte balance [20]. Advancements in wearable sensor technology have facilitated the real-time monitoring of the sweat composition of individuals, aiding in the assessment of physical performance and health [22,23]. Wearable sweat sensors are becoming more popular among athletes, soldiers, and people who need to watch for dehydration.

Urine is a liquid byproduct of metabolism that is excreted by the kidneys. It is a key fluid in the assessment of renal function, hydration levels, and metabolic disorders. Urine contains metabolites, proteins, and waste products. Urinalysis can reveal the presence of various substances, including electrolytes, proteins, and metabolic waste products, providing valuable information for diagnosing and monitoring numerous health conditions [24,25]. Portable urine sensors enable early kidney disease detection and drug monitoring.

Tears are produced by the lacrimal glands and play a crucial role in maintaining ocular health. They contain proteins and electrolytes that can provide valuable information about ocular and systemic diseases [26,27]. Recently, nanosensors have been introduced which are integrated into contact lenses, allowing for non-invasive glucose and cortisol monitoring in patients [28,29]. The analysis of individuals’ tear composition is a potential non-invasive approach for disease detection and monitoring.

Interstitial fluid (ISF) surrounds cells and contains nutrients, waste products, and biomarkers, making it an ideal candidate for continuous health monitoring. It closely resembles plasma in composition and serves as a promising medium for the continuous monitoring of biomarkers such as glucose, offering a less invasive alternative to blood sampling [30,31]. Microneedle sensors enable minimally invasive ISF analysis for real-time glucose and lactate monitoring, which is critical in the management of diabetes and metabolic disease.

The analysis of these body fluids through advanced sensing technologies, including microsensors and nanosensors, has revolutionized disease detection, personalized medicine, and real-time health monitoring. These innovations enable the detection and quantification of various biological, chemical, and physical parameters, facilitating early intervention and improved patient outcomes.

## 3. Fundamentals and Classification of Microsensors and Nanosensors

### 3.1. Definition and Working Principles

Nanosensors and microsensors are miniaturized devices that detect and quantify biochemical, physical, or chemical changes in biological samples. These sensors operate at the micro and nano scales, offering high sensitivity, selectivity, and real-time monitoring capabilities.

While nanosensors and microsensors differ primarily in their physical dimensions, it is important to note that the performance of a sensor is influenced by a combination of factors including the sensor’s architecture, material composition, surface chemistry, and transduction mechanisms. Nanosensors, typically characterized by dimensions below 100 nm, often exhibit enhanced surface/volume ratios that can increase their sensitivity due to there being more active sites for analyte interaction. Additionally, unique quantum and size-dependent effects at the nano scale can further improve the detection limits in certain applications. However, the sensitivity and selectivity of these sensors are not solely dependent on the sensor’s size; they are also heavily influenced by the specificity of their recognition elements and their efficiency of signal transduction. Moreover, the capability of a sensor for real-time monitoring is largely dictated by its response time and data processing techniques rather than its size. Microsensors, with dimensions ranging from micrometers to millimeters, can offer advantages such as easier fabrication, integration with electronics, and robustness. Therefore, the choice between micro- and nanosensors should be guided by the specific application requirements, balancing factors such as sensitivity, selectivity, fabrication complexity, and intended deployment environment.

The working principle of nanosensors and microsensors is typically based on the interaction between the target analyte and a functionalized sensing surface, which should lead to a measurable signal transduction [32,33,34].

The signal transduction mechanism varies depending on the type of sensor but generally falls into one of four primary categories:

1. Electrochemical—measures electrical signals (e.g., current, voltage, impedance) produced by the interaction of the analyte with the sensor [35];
2. Optical—uses fluorescence, absorbance, or other light-based methods to detect biomolecular interactions [17];
3. Piezoelectric—detects changes in mass or mechanical forces by monitoring shifts in the resonant frequency [36];
4. Field-effect transistor (FET)-based—uses semiconducting materials to modulate the sensor’s electrical conductivity in response to analyte binding [37].

These transduction mechanisms enable the real-time detection of biomolecules, making nanosensors essential in disease diagnostics, drug monitoring, and personalized medicine.

### 3.2. Classification Based on Transduction Mechanism

A wide range of transduction mechanisms are used in miniaturized biosensors, including electrochemical, optical, piezoelectric, and field-effect transistor (FET)-based approaches. While these techniques are well-established, recent developments have demonstrated trends toward specific use-cases that are based on the operational principles and integration compatibility of specific sensor types.

#### 3.2.1. Electrochemical Sensors

Electrochemical nanosensors detect biomolecules by measuring electrical signals such as the current, voltage, or impedance of the analyte [35]. These sensors are widely used for glucose monitoring, lactate detection, and disease biomarker quantification. For instance, the voltammetry technique, based on graphene oxide, was used in glucose biosensors developed by Chen et al. (2024) for glucose monitoring in athletes. The detection limit of glucose in blood serum was determined to be 0.046 μM [38]. Another example is the ultra-small wearable flexible biosensor for the analysis of glucose, lactate, Na+, and K+ in sweat that was presented in 2022 by Zhang et al. The whole system mainly includes flexible electrodes and a printed circle board (PCB) and is used to perform electrochemical signal processing [39].

Electrochemical sensors remain the most widely adopted form of wearable and implantable biosensors due to their compact form factor, low power consumption, and compatibility with microfabrication techniques. Their ability to function in complex biological matrices (e.g., sweat, interstitial fluid) with relatively simple signal processing methods has made them a dominant platform for continuous monitoring applications.

#### 3.2.2. Optical Sensors

Optical sensors detect analytes by measuring changes in fluorescence, absorbance, or Raman scattering [40]. These sensors are particularly useful for detecting low-concentration biomarkers in saliva, urine, and tears. An example of the application of such a mechanism is an immunoassay for cancer biomarker (prostate-specific antigen) detection that is based on surface-enhanced Raman scattering nanosensors. This sensor, designed using gold nanoparticles, was employed by Grubisha et al. in 2003 [41]. The PSA detection limit was determined to be 1 pg/mL in human serum. The optical mechanism has also been applied in a wearable fluorescence nanosensor for the monitoring of glucose in sweat through a smartphone (Kansay et al. 2024) [42]. The sensor was developed for the real-time measurement of an individual’s sweat glucose concentration via a smartphone readout, and was composed of a fluorescent nanosensor probe based on boric acid-functionalized and heteroatom-doped carbon quantum dots (CQDs) that were embedded in paper-based analytical devices and integrated with a hydrophilic cotton thread-based microfluidic channel. The limit of detection value for the in situ measurement of the glucose concentration of sweat was from 1.40 to 2.00 μM for this sensor.

Optical sensors offer significant advantages for intra-body and non-invasive sensing, particularly in applications such as photoplethysmography, fluorescence-based glucose monitoring, and optical pH sensing. However, their integration into ingestible or long-term implantable devices is limited by challenges regarding light transmission, alignment, and potential signal loss in turbid biological tissues.

#### 3.2.3. Piezoelectric Sensors

Piezoelectric sensors work by detecting mass or force changes through shifts in resonant frequency [36]. These sensors are widely used in wearable biosensing applications for monitoring cardiovascular and respiratory conditions. An illustrative use of the piezoelectric effect can be seen in the work by Cao et al. (2024) [43]. A flexible piezoelectric nanogenerator-based biosensor was recently developed for the real-time monitoring of the chloride levels and pH of sweat. The system is powered by piezoelectric ceramics, and a microchannel structure guides sweat to the storage chamber, where color changes, detected by sensors, reflect the amount of chloride ions and pH values that are detected. Near-field communication (NFC) technology enables wireless data transmission to mobile devices. Another example is a piezoelectric quartz crystal sensor that was recently designed by Saleh et al. (2025), which was functionalized with antibodies to detect COVID-19 antigens [44].

Piezoelectric and acoustic wave-based sensors have shown promise in detecting mechanical changes and mass loading, particularly for the purpose of pathogen or virus detection. However, they often require more complex electronics and are less robust under dynamic physiological conditions.

#### 3.2.4. Field-Effect Transistor (FET)-Based Sensors

FET-based nanosensors utilize semiconducting materials (such as graphene, silicon nanowires) to modulate the electrical conductivity of an analyte upon its binding [45]. These sensors offer the ultrasensitive, label-free detection of biomolecules. An example of this group of sensors is a graphene-FET sensor designed to detect an Alzheimer’s disease protein biomarker. The GFET biosensor was developed by Bungom et al. (2021) [46] for the detection of clusterin, a prominent protein biomarker of Alzheimer’s disease. The limit of detection of the biosensors is only 4 fM. A representative application of this mechanism can be also found in a work by Premanode et al. (2007), in which a biosensor was used for the monitoring of creatinine and urea [12]. An ion-sensitive field-effect transistor with associated circuitry demonstrated a linear relationship between urea and creatinine in the range of 0–200 and 0–20 mM, respectively.

FET-based sensors (particularly those based on graphene or other nanomaterials) are emerging as highly sensitive platforms that are capable of label-free detection. Their ability to convert biochemical interactions into electrical signals without intermediate labeling steps offers unique advantages for real-time monitoring, although their fabrication and long-term stability are still under investigation.

A comparison of these technologies is provided in Table 1, which outlines key parameters such as their sensitivity, biocompatibility, integration potential, cost, and real-time performance. This roadmap offers perspective regarding which transduction mechanisms are likely to be most impactful in the development of next-generation wearable, implantable, or ingestible biosensing devices.

### 3.3. Classification Based on Materials Used

#### 3.3.1. Carbon-Based Sensors

Carbon nanomaterials, including carbon nanotubes (CNTs), graphene, and graphene oxide (GO), have emerged as highly attractive materials for sensor applications due to their exceptional electrical conductivity, high surface area, mechanical strength, and chemical stability. Their unique physicochemical properties facilitate efficient electron transfer and allow for the immobilization of biomolecules, which enhance the sensitivity and selectivity of biosensors. In the context of body fluid monitoring, carbon nanomaterials have been widely integrated into electrochemical and optical nanosensors for the detection of key biomarkers such as glucose, lactate, uric acid, and various proteins. Moreover, their compatibility with flexible electronics makes them ideal candidates for use in next-generation wearable and implantable diagnostic devices. Carbon-based materials exhibit high conductivity, flexibility, and biocompatibility, making them ideal for wearable and implantable biosensors [47,48,49]. Carbon-based materials have been widely utilized in the fabrication of various sensors that were employed for the analysis of body fluids, for instance, graphene was used in a glucose biosensor [38], carbon nanotubes in uric acid and dopamine sensors [50], and carbon black in a urine sensor [51].

#### 3.3.2. Metal Nanoparticles

Metal nanoparticles, including gold (Au), silver (Ag), and platinum (Pt) nanoparticles, are widely utilized in sensor technologies due to their unique optical, electronic, and catalytic properties. Their high surface-area/volume ratio and variable size allow for enhanced interaction with target analytes, which improves the sensitivity and specificity of biosensors. In body fluid monitoring, metal nanoparticles are commonly incorporated into electrochemical, colorimetric, and surface-enhanced Raman spectroscopy (SERS) sensors, enabling the detection of biomarkers such as glucose, DNA, and proteins at low concentrations. Additionally, their biocompatibility and the ease of their functionalization with biomolecules make them ideal candidates for the development of personalized and real-time diagnostic devices [52]. An example of the utilization of gold nanoparticles is an immunoassay for cancer biomarker detection that is based on surface-enhanced Raman scattering nanosensors (Grubisha et al., 2003) [41]. The wide applicability of silver nanoparticles in optical and electrochemical biosensors in point-of-care treatment was discussed by Beck at al. [53].

#### 3.3.3. Quantum Dots and Nanowires

Quantum dots (QDs) are semiconductor nanoparticles with unique optical properties, such as size-dependent fluorescence and high photostability, that make them ideal for use in biosensor applications. Due to their well-defined size and shape, quantum dots exhibit strong and narrow emission spectra, which allow for the precise, simultaneous detection of multiple biomarkers. In the context of body fluid analysis, quantum dots are often used in fluorescence-based sensors for detecting biomarkers such as proteins, DNA, and small molecules. Their high surface area also facilitates functionalization with targeting ligands or antibodies, which enhances the selectivity and sensitivity of sensors for real-time, non-invasive diagnostics [54]. Silicon nanowires (SiNWs) are one-dimensional nanomaterials with remarkable electrical, optical, and mechanical properties that make them highly suitable for sensor applications. Due to their high surface-area/volume ratio and excellent conductivity, nanowires are particularly effective in enhancing the sensitivity and performance of biosensors. In body fluid analysis, nanowires are commonly integrated into electrochemical and field-effect transistor (FET)-based sensors for the detection of low concentrations of biomarkers such as glucose, lactate, and various proteins. Their ability to form conductive networks and the ease of their functionalization with biomolecules make them ideal candidates for the development of sensitive, real-time diagnostic devices for personalized healthcare and continuous monitoring [55,56,57]. Quantum dots were utilized in a wearable fluorescence nanosensor for monitoring glucose (Kansay et al. 2024) [42], while nanowires were used in ultrasensitive, label-free, and real-time silicon field-effect transistors for PSA detection [58].

#### 3.3.4. Polymer-Based Sensors

Conducting polymers are a class of organic polymers that exhibit intrinsic electrical conductivity due to their conjugated π-electron backbone. Materials such as polyaniline (PANI), polypyrrole (PPy), and polythiophene (PT) have attracted significant interest in sensor development owing to their excellent electrical properties, environmental stability, and chemical functionality. These polymers facilitate efficient electron transfer and can be functionalized with biorecognition elements, which makes them ideal for transducing biochemical interactions in electrochemical sensors. In the context of body fluid analysis, conducting polymers are often employed as active sensing layers or as components of nanocomposites, enabling the sensitive and selective detection of biomarkers such as glucose, urea, and lactate. Their compatibility with flexible substrates also makes them promising materials for wearable and implantable biosensors [59]. PANI was implemented into a wearable electrochemical sensor for pH sweat monitoring [60], polydimethylsiloxane (PDMS) was used in a flexible piezoelectric nanogenerator-based biosensor for the real-time monitoring of the chloride levels and pH of sweat [43], and PPy is widely applied in the early diagnosis of colorectal cancer [61].

Particular attention should be given to molecularly imprinted polymers (MIPs). MIPs are synthetic polymeric materials that are engineered to possess highly specific recognition sites for target molecules. These recognition sites are created through a template-assisted polymerization process, wherein the target analyte (template molecule) is embedded in a monomer matrix and subsequently removed, leaving behind complementary cavities in terms of their shape, size, and functional groups. This “molecular memory” allows MIPs to selectively bind to their template molecules, much like natural antibodies or receptors, but with enhanced chemical and thermal stability. Due to their high specificity, low cost, and robustness, MIPs have emerged as promising alternatives to biological recognition elements in sensor design, particularly for applications in diagnostics, environmental monitoring, and pharmaceutical analysis [62,63]. Their integration into electrochemical, optical, and mass-sensitive sensors has facilitated the detection of a wide range of analytes, including drugs, hormones, proteins, and even pathogens [64,65,66]. An example of the application of MIPs in the analysis of body fluids is the molecularly imprinted fluorescence sensor chip for lactate measurement that was developed by Wusiman et al. (2024) [67].

## 4. Construction Solutions for Microsensors and Nanosensors

### 4.1. Microfluidic-Based Sensors

Microfluidic-based sensors are analytical devices that incorporate micro-scale fluidic systems to manipulate small volumes of biological or chemical fluids (typically in the microliter to nanoliter range) for sensing applications. These platforms integrate microchannels, chambers, and detection components on a single chip, allowing for precise control, mixing, and the analysis of body fluids such as blood, saliva, sweat, or urine. Microfluidic sensors are particularly advantageous in point-of-care diagnostics due to their low sample and reagent requirements, high surface/volume ratio, rapid response time, and potential for multiplexed detection. Their miniaturized nature facilitates integration with wearable or portable devices for continuous, real-time health monitoring. Recent advances in microfabrication and materials science have enabled the development of highly sensitive microfluidic-based sensors that can detect biomarkers for diseases such as cancer, diabetes, and infectious conditions with high specificity. These sensors are often coupled with optical, electrochemical, or piezoelectric transduction mechanisms, which provides versatile platforms for both qualitative and quantitative analysis in clinical and biomedical research [68,69].

In the 19801990s, microfluidics began as an extension of microelectromechanical systems (MEMS). Scientists started adapting MEMS technologies to manipulate fluids on a micro scale. One of the earliest practical devices to use microfluidics for analysis was the Miniaturized Total Analysis System (µTAS), a concept introduced in the early 1990s. Andreas Manz is widely credited as one of the pioneers of microfluidics. He introduced the µTAS concept in the early 1990s, aiming to integrate sample handling, separation, and detection on a single chip—the foundation of many modern microfluidic sensors [70]. By the late 1990s to early 2000s, microfluidic systems began to be integrated with electrochemical, optical, and piezoelectric sensors, which marked the emergence of microfluidic-based sensors for biomedical applications. These integrations enabled the fabrication of lab-on-a-chip (LOC) and point-of-care (POC) systems that are used today in diagnostics, environmental monitoring, and wearable devices.

**Figure 2:**
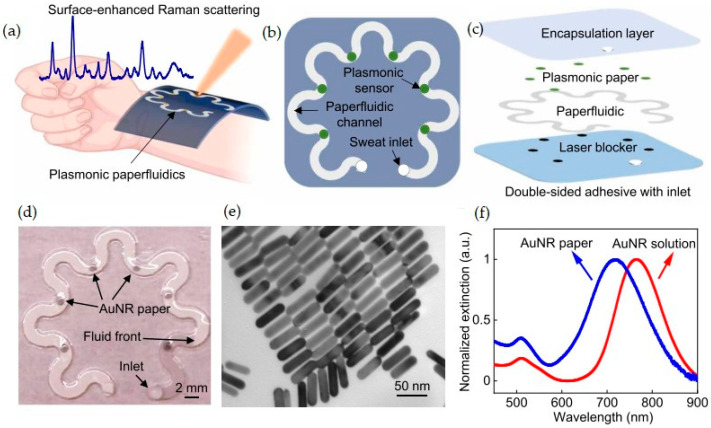
Wearable plasmonic paper-based microfluidic system for sweat analysis, developed by Mogera et al. (2022). (**a**): scheme illustration of sensor, (**b**): top view and (**c**): layered view of the pa-perfluidic device, (**d**): assembled device, (**e**): TEM image of gold nanorods (AuNR), (**f**): spectra of AuNR. Reprinted from reference [71].

As an example of a microfluidic sensor, we present the wearable plasmonic paper-based microfluidic system for sweat analysis that was developed by Mogera et al. [71]. This wearable device integrates paper-based microfluidics with plasmonic sensors to enable the continuous and simultaneous quantitative analysis of the sweat loss, sweat rate, and metabolites, such as uric acid, of an individual. The system utilizes surface-enhanced Raman spectroscopy (SERS) for sensitive detection. The soft, thin, flexible, and stretchable device, laminated on a wearer’s wrist for sweat collection, is presented in Figure 2a The wearable plasmonic sweat sensors comprise several functional layers, including a double-sided adhesive, a laser blocker, a paper microfluidic layer, plasmonic sensors, and a top encapsulation layer (Figure 2b,c). Figure 2d presents an assembled paper-fluidic device. The sensor was designed based on gold nanorods (AuNR paper), as shown in Figure 2e. SERS detection (Figure 2f) allows for the highly sensitive detection of uric acid.

Microfluidic devices have also been developed to detect cancer biomarkers in body fluids, utilizing technologies such as electrochemical sensors, surface-enhanced Raman spectroscopy (SERS) biosensors, and immunosensors. These devices offer the precise control of fluid dynamics and enhanced interaction between the sensor and analyte, which improve their detection limits and specificity [72].

**Figure 3:**
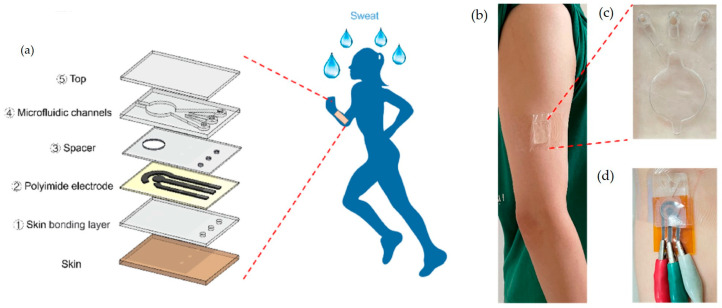
Wearable sensors designed by Zhang et al. (2025) [73], reprinted with permission from Elsevier. (**a**): Layered view of the wearable microfluidic sensor; (**b**): picture of the microfluidic chip affixed to the arm, (**c**): an image of a microfluidic device collecting sweat, (**d**): a diagram of microfluidic device measurement in electrochemical workstation.

Microfluidic sensors have been widely utilized in wearable technologies [11]. As an example, we cite a wearable microfluidic biosensor for the simultaneous, real-time detection of biomarkers such as uric acid, dopamine, and tyrosine in sweat that was developed by Zhang et al. (2025) [73]. The device has an enhanced sweat collection efficiency and provides the simultaneous monitoring of multiple analytes. The schematic representation is reprinted from Elsevier, Amsterdam, Netherlandsand presented in Figure 3.

### 4.2. Lab-on-a-Chip (LoC) Systems

The pioneering work on lab-on-a-chip technology was that by S.C. Terry, J.H. Jerman, and J.B. Angell which was first presented in their 1979 paper titled “A Gas Chromatographic Air Analyzer Fabricated on a Silicon Wafer”, which was published in *IEEE Transactions on Electron Devices* [74]. This paper introduced a miniaturized gas chromatograph integrated onto a silicon wafer, marking a significant milestone in the development of microfabricated analytical devices. A lab-on-a-chip (LOC) is a miniaturized device that integrates multiple laboratory functions onto a single chip which ranges from a few square millimeters to a few square centimeters in size. These devices are capable of handling extremely small fluid volumes, down to less than picolitres, and are designed to automate and execute complex biochemical operations, such as chemical synthesis, DNA sequencing, and biochemical analysis, which traditionally require an entire laboratory setup. The miniaturization inherent in LOC systems offers several advantages, including reduced reagent consumption, faster analysis times, and the potential for high-throughput screening. By leveraging microfluidic technologies, LOC devices facilitate the precise control and manipulation of fluids at the micro scale, enabling the efficient and rapid processing of biochemical reactions [75,76,77]. Lab-on-a-chip devices are a subset of microelectromechanical systems (MEMS) devices.

**Figure 4:**
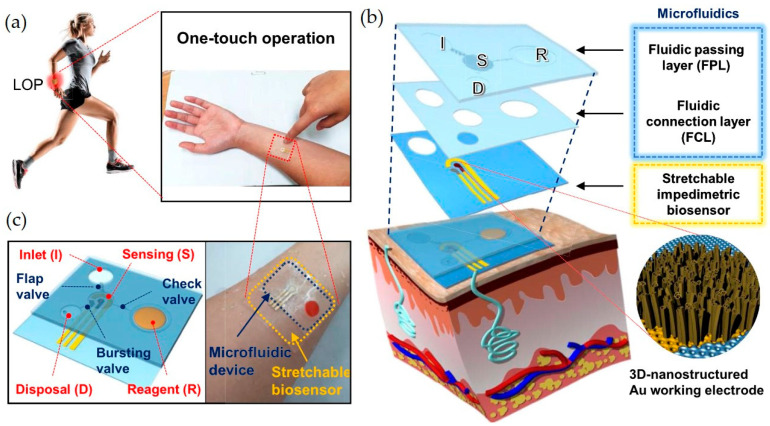

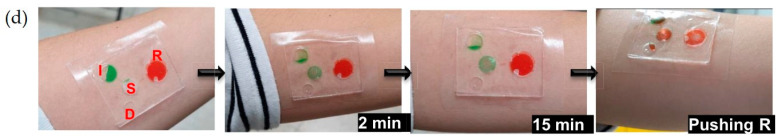
Lab-on-a-chip wearable sensor for cortisol detection in sweat, designed by Lee at al. (2020). (**a**): A schematic diagram of the LOP platform for wearable cortisol detection, (**b**): a schematic view of the patch and a picture of the sensor attached to a forearm, (**c**): a layered view of the lab-on-a-chip wearable sensor—the top layer is the fluidic layer with the chambers and channels, the middle layer is the connecting layer between the chambers and a skin, and the bottom layer contains a stretchable impedimetric immunosensor with a 3D-nanostructured Au working electrode, (**d**): demonstration of microfluidic device while it is attached to an arm. Reprinted from reference [78] with permission from Elsevier.

An example of a LOC sensor is the wearable Lab-on-a-Patch (LOP) which was designed by Lee at al. (2020) to detect cortisol in sweat [78]. This conformable, wearable LOC platform integrates a stretchable, label-free, impedimetric biosensor with a stretchable microfluidic device. A microfluidic device integrated with an electrochemical biosensor in a patch is attached to human skin. The microfluidic device collects a sweat sample, transports the sample to a sensing chamber that contains a stretchable impedimetric immunosensor, simultaneously delivers a reagent and washes the sensing chamber, then disposes of the waste. It enables the on-body detection of cortisol levels in sweat, utilizing three-dimensional nanostructured gold electrodes to obtain high sensitivity. Electrochemical detection allows for the detection of the pM levels of cortisol in sweat. Figure 4 presents a schematic representation of a LOC sensor, which was reprinted with the permission of Elsevier, Amsterdam, Netherlands.

An additional example of sensors that can be cited is colorimetric sensors. The first example of this type of sensor is a lab-on-a-chip optical sensor for salivary glucose measurement that was designed by Jung et al. (2017) [79]. This LOC-based optical sensor utilizes a glucose oxidation reaction and the enzymatic colorimetric method to measure the glucose levels in saliva. It offers a non-invasive alternative to blood glucose monitoring for diabetes management. Another notable example is a wearable microfluidic sweat chip for glucose and pH monitoring that was presented by Liu et al. (2023). This wearable microfluidic chip collects sweat during exercise and utilizes colorimetric sensors to detect the glucose concentration and pH levels in the collected sweat. The device allows for the non-invasive, real-time monitoring of metabolic parameters in athletes [80].

Current systems face several technical limitations, including the sensor’s stability over time, power requirements, and the need for bulky external instrumentation for signal readout and data processing. This is particularly relevant in lab-on-a-chip (LOC) technologies, which, while highly miniaturized in terms of fluid handling, often still depend on off-chip detectors, light sources, or control units, especially in optical and electrochemical systems.

For instance, many optical biosensors, such as those based on surface plasmon resonance or fluorescence, still require external light sources, lenses, and photodetectors, making full integration difficult. This significantly limits their application in implantable or ingestible formats. Similarly, electrochemical LOC devices may rely on benchtop potentiostats, although recent advancements are addressing this gap through on-chip signal amplification and the use of wireless modules and integrated microcontrollers [81,82].

Efforts to overcome these limitations include the development of fully integrated systems-on-a-chip (SoC) or systems-in-a-package (SiP) platforms, where fluidic handling, sensing, power, and communication are combined into a single, miniaturized unit. For example, Bandodkar et al. (2019) introduced a battery-free skin-interfaced system that performs electrochemical, colorimetric, and volumetric sweat analysis without external components [83]. While true “implantable” or “ingestible” lab-on-a-chip systems are still emerging, significant progress is being made in micro-optoelectromechanical systems (MOEMS) and flexible electronics that enable such possibilities.

Thus, while lab-on-a-chip platforms are mature in terms of microfluidic manipulation, the real frontier lies in full system miniaturization and autonomy, which is essential for future wearable, implantable, or ingestible diagnostics.

### 4.3. MEMS-Based Sensors

Micro-electromechanical systems (MEMS)-based sensors are miniaturized devices that integrate mechanical and electrical components at the micro scale to detect and measure various physical, chemical, or biological phenomena. These sensors typically consist of mechanical microstructures, microsensors, microactuators, and microelectronics, which are all integrated onto a single silicon chip. The small size of MEMS sensors allows for reduced power consumption and their cost-effective mass production through batch fabrication techniques.

MEMS sensors operate by transducing mechanical signals—such as pressure, acceleration, or force—into electrical signals that can be processed and analyzed. This transduction is achieved through various sensing mechanisms, including capacitive, piezoresistive, piezoelectric, and thermal methods. The choice of mechanism depends on the specific application and desired sensor characteristics [84,85]. Due to their compact size, low power consumption, and high performance, MEMS-based sensors have found widespread applications across multiple fields. They are extensively used in consumer electronics (accelerometers and gyroscopes in smartphones), automotive systems (airbag deployment sensors), healthcare (blood pressure monitors), and environmental monitoring (gas sensors) [86].

**Figure 5:**
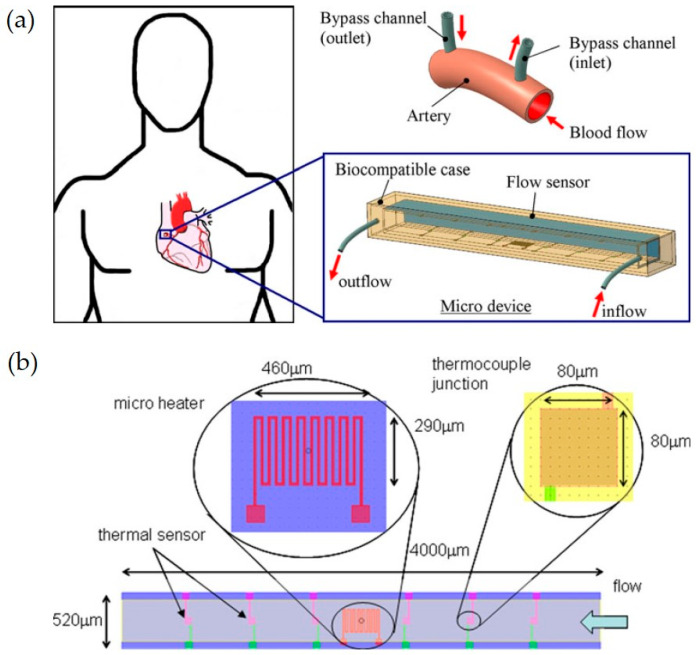
Schematic representation of MEMS sensing device. (**a**): diagram of MEMS-based implantable flow sensing device application, (**b**): model of mass flow sensor. Content is licensed by Springer Nature Customer Service Center GmbH [87].

A MEMS-based micro flow sensor for arterial blood flow measurement, presented by Mistry et al. (2012), is a notable example of the utilization of MEMS technology in body fluid analysis. This study presents the design and simulation of a thermal MEMS-based micro-flow sensor designed to measure arterial blood flow. In Figure 5, a schematic representation of an implantable MEMS sensing device is presented, with its reprint having been licensed by Springer Nature [87]. The sensor operates by detecting convective heat transfer variations caused by blood flow, which enables the precise measurement of flow rates as low as 10 µL/min.

### 4.4. Integration with Smart Wearables and Implantables

Wearable and implantable nanosensors are essential in advancing personalized healthcare due to enabling the continuous monitoring of physiological parameters. While both technologies aim to enhance health monitoring, they differ in their design, application, and integration with the human body [88]. Wearable nanosensors offer a non-invasive means for real-time health monitoring, which makes them suitable for a broad range of applications, while implantable nanosensors provide continuous, accurate data from within the body, which is essential for managing chronic conditions and critical health parameters. The choice between wearable and implantable nanosensors depends on the specific healthcare application, the required monitoring precision, and considerations of user comfort and safety [89].

Wearable nanosensors are externally applied devices that are designed to detect physiological signals through the skin or other non-invasive means. They are typically flexible, lightweight, and comfortable, facilitating the real-time monitoring of various health metrics.

**Figure 6:**
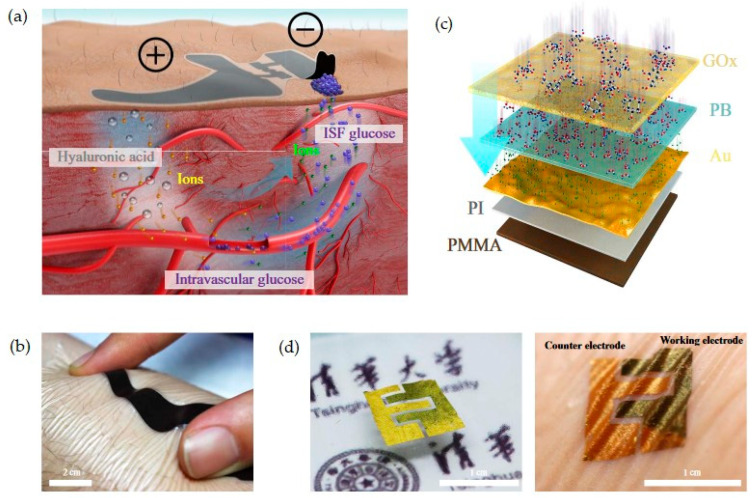
Skin-like biosensor for glucose monitoring developed by Chen et al. (2017). (**a**): schematic representation of the sensor operation, (**b**): ultra-thin skin-like biosensor, (**c**): layered construction of biosensor, (**d**): electrochemically deposited dual electrodes of the biosensors and skin-adherent biosensor. Reprinted from Skin-like biosensor system via electrochemical channels for non-invasive blood glucose monitoring, *Sci. Adv.* 2017 [90].

Some of the most noteworthy contributions to the field of wearable sensors that are presented in the literature include the skin-like biosensor for glucose monitoring developed by Chen et al. (2017) [90] and the tear glucose sensor developed by Kownacka et al. (2018) [91]. The skin-like biosensor is a non-invasive system for intravascular blood glucose monitoring. The system consists of a flexible biocompatible paper battery and ultra-thin skin-like biosensors. The device drives intravascular blood glucose out of the vessel and transports it to the skin surface. The ultra-thin nanostructured biosensor fully absorbs and measures the glucose in blood with high sensitivity. A reprint from the work by Chen et al. is provided in Figure 6 [90].

**Figure 7:**
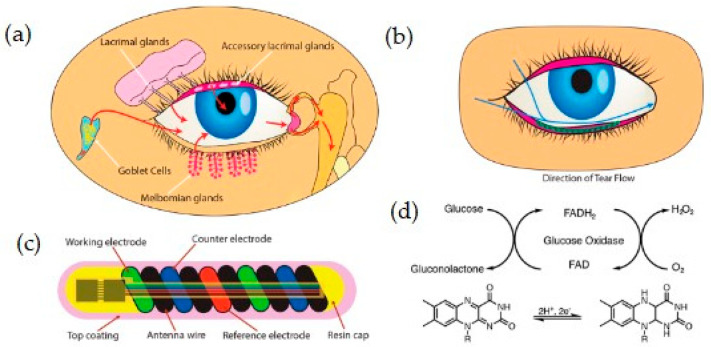
NovioSense minimally invasive tear glucose sensor. (**a**): Schematic representation of the tear fluid production, (**b**): direction of the tear flow, (**c**): design and structure of the final product with electronic components, (**d**): mechanism of glucose detection. Reprinted from Clinical evidence for use of a noninvasive biosensor for tear glucose as an alternative to painful finger-prick for diabetes management utilizing a biopolymer coating, *Biomacromolecules* 2018 [91].

The tear glucose sensor, named NovioSense Glucose Sensor, utilized by Kownacka et al. (2018) in a clinical trial is worn under the lower eye and designed to continuously measure glucose levels in the basal tear fluid [91]. This sensor is based on an electrochemical detection mechanism. The research showed a good correlation to blood glucose values and excellent linearity in the concentration range from 0−20 mM. A schematic representation of the biosensor is presented in Figure 7.

Implantable nanosensors are devices that are embedded within the body to provide the continuous, real-time monitoring of internal biochemical and physiological processes. They offer the advantage of accessing interstitial fluids and tissues directly, which enables precise measurements [92]. They have been explored for their potential in continuous monitoring systems, which is due to their ability to offer insights into chronic conditions and aid in timely medical interventions.

**Figure 8:**
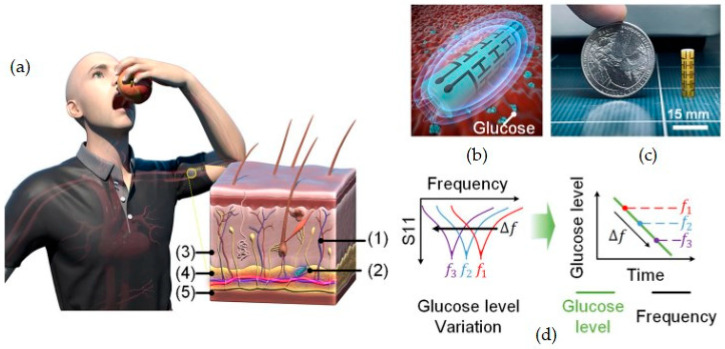
Subcutaneous implant glucose sensor. (**a**): Scheme of implantable sensor for blood glucose level monitoring; (1) blood capillary, (2) electromagnetic sensor, (3) dermis, (4) subcutaneous fat, (5) muscle tissue; (**b**,**c**): implant sensor; (**d**): sensor frequency trend. Reprinted from Subcutaneously implantable electromagnetic biosensor system for continuous glucose monitoring. *Sci. Rep.* 2022 [93].

As an example of the application of an implantable sensor, we mention an electromagnetic-based sensor for glucose monitoring that was proposed by Kim et al. (2022) [93]. The sensor can be subcutaneously implanted and is capable of tracking minute changes in dielectric permittivity owing to changes in the blood glucose level. A schematic representation and brief information about the sensor can be found in Figure 8.

## 5. Targeted Analytes for Microsensors and Nanosensors for Body Fluid Monitoring

The analysis of body fluids can be divided into that which uses targeted biomarkers and that which involves drug monitoring. Biomarkers are measurable biological indicators that reflect normal physiological processes, pathological changes, or responses to therapeutic interventions. They are commonly found in body fluids such as blood, saliva, sweat, urine, tears, and interstitial fluids, and play a key role in disease diagnosis, disease prognosis, and treatment monitoring. In addition to endogenous biomarkers, exogenous compounds such as therapeutic drugs are also important analytes for monitoring treatment adherence and pharmacokinetics. The development of microsensors and nanosensors has enabled the sensitive, real-time, and often non-invasive detection of a wide spectrum of analytes. These can be broadly grouped into the categories of metabolites, electrolytes, proteins, hormones, nucleic acids, gases, and drugs, each of which provides specific insights into an individual’s health status [94,95].

### 5.1. Metabolites

Metabolites are small molecules involved in or produced by metabolic processes and serve as important indicators of physiological and pathological states. In body fluids such as blood, urine, saliva, sweat, and interstitial fluid, metabolites provide valuable information on cellular activity, energy metabolism, and organ function [96]. Commonly monitored metabolites are glucose, lactate, urea, creatinine, and uric acid, each of which is associated with specific metabolic pathways and clinical conditions. In this section, we define the most frequently analysed metabolites and provide a comparison of microsensors and nanosensors that utilize different construction solutions which have been presented so far in literature (Table 2).

Glucose is one of the most critical biomarkers monitored in body fluids due to its significant role in energy metabolism and its relevance in diagnosing and managing diabetes [16,97]. Traditional glucose monitoring relies on invasive blood sampling; however, advancements in microsensor and nanosensor technologies have enabled non-invasive or minimally invasive monitoring through the analysis of alternative body fluids such as sweat, saliva, tears, and interstitial fluid. Continuous glucose monitoring systems that use wearable sensors, particularly those that target interstitial fluid, have revolutionized diabetes care by providing real-time data, reducing the need for frequent finger-pricks, and improving glycaemic control. Recent developments in electrochemical and optical nanosensors [17] have further enhanced their sensitivity, specificity, and user comfort, supporting the shift toward personalized and preventive healthcare [15,98]. The application of thin-film holographic sensors with optical detection has lowered the limit of detection of glucose in blood serum to 3 mM [99]. With the use of electrochemical wearable microneedle-based arrays, it is possible to detect glucose in interstitial fluid with a limit of 7.44 mM [100]. Other wearable sensors can also be used for glucose monitoring in sweat. Microsensors and nanosensors that are used for glucose analysis in body fluids are compared in Table 2.

While enzymatic electrochemical sensors using glucose oxidase have long dominated the market due to their specificity and simplicity, current research is shifting toward non-invasive or minimally invasive approaches and enhancing the stability of sensors for continuous monitoring. Emerging strategies include the use of fluorescent nanomaterials (e.g., quantum dots, carbon nanodots) in optical detection, FET-based biosensors for label-free sensing, and microneedle patches that access interstitial fluid painlessly [83]. A major trend is the development of non-enzymatic sensors that are based on nanostructured materials (e.g., metal–organic frameworks, graphene composites), which offer improved long-term stability and resistance to interference from other analytes. Despite the significant progress in this field, key challenges persist. These include the calibration drift in continuous glucose monitors (CGMs), biofouling and the loss of sensitivity in long-term implantable systems, the lag time between the analysis of interstitial fluid glucose and blood glucose concentrations, non-specific binding and cross-reactivity in complex biological matrices, and miniaturization with integrated data transmission for wearables and implants. Addressing these issues requires advances not only in sensor chemistry and materials science but also in on-chip data processing, power efficiency, and real-time calibration techniques.

Lactate is a key metabolic biomarker that reflects the balance between aerobic and anaerobic metabolism, making it a valuable indicator of physiological stress, tissue hypoxia, and metabolic disorders. Elevated lactate levels are commonly associated with conditions such as sepsis, shock, and intense physical exertion. Traditionally measured in blood, lactate can also be monitored through alternative body fluids, including sweat [101], saliva, and interstitial fluid [102], offering less invasive assessment options. The integration of lactate-sensitive microsensors and nanosensors into wearable and implantable platforms has enabled real-time monitoring, which is particularly useful for athletes, critical care patients, and individuals with metabolic diseases. Among the existing wearable sensors for lactate monitoring, we can distinguish wearable microneedle arrays [100], eyeglass sensors [101], and temporary transfer tattoos [103]. Recent innovations in electrochemical and enzymatic biosensors have significantly improved the accuracy and responsiveness of lactate detection, facilitating continuous health monitoring and early intervention in both clinical and athletic settings [104,105]. Exemplary microsensors and nanosensors that incorporate electrochemical detection are compared in Table 2.

Uric acid is the product of purine metabolism and serves as an important biomarker for conditions such as gout, hyperuricemia, cardiovascular diseases, and renal dysfunction. Its abnormal accumulation in the body can lead to crystal formation in the joints or kidneys, contributing to inflammation and organ damage [106]. The development of nanosensors and microsensors, particularly electrochemical biosensors that incorporate materials like carbon nanotubes, graphene, and metal nanoparticles, has improved the sensitivity, selectivity, and portability of uric acid detection. The integration of these sensors into wearable platforms or lab-on-a-chip (LOC) systems offers promising avenues for continuous and point-of-care monitoring, contributing to the early diagnosis and personalized management of metabolic and renal disorders [107,108]. Uric acid is traditionally measured in the blood, yet recent technological advances have enabled its detection in alternative body fluids such as intestinal fluid [109], saliva [110], and sweat [111], facilitating the non-invasive monitoring of uric acid. Microsensors and nanosensors that are used for the determination of uric acid in various body fluids are compared in Table 2.

Creatinine is a metabolic byproduct of muscle activity and a well-established biomarker for evaluating renal function. Its concentration in body fluids is commonly used to assess the glomerular filtration rate (GFR), aiding in the diagnosis and monitoring of chronic kidney disease (CKD) and other renal disorders. Recent advances in electrochemical biosensors, which are often based on piezoelectrics, have demonstrated high sensitivity and a low detection limit in creatinine detection (1 × 10^−5^ mM) [112]. These sensors are increasingly being integrated into wearable and point-of-care (POC) diagnostic systems, supporting continuous kidney function monitoring and early intervention strategies [113].

Cholesterol is a vital lipid molecule that is involved in the formation of the cellular membrane structure, hormone synthesis, and metabolic processes. However, abnormal cholesterol levels are strongly associated with cardiovascular diseases, including atherosclerosis, heart attacks, and strokes. Traditionally done through blood tests, the monitoring of cholesterol levels is critical for assessing cardiovascular risk and guiding therapeutic interventions. Advances in microsensor and nanosensor technologies have enabled the development of electrochemical and optical biosensors that are capable of detecting cholesterol in the blood and, potentially, in alternative fluids such as sweat and tears, offering less invasive monitoring approaches. These sensors, particularly when integrated into wearable microneedles [114] or smart contact lenses [29], facilitate real-time lipid profiling, supporting early diagnosis, lifestyle monitoring, and personalized treatment strategies for patients who are at risk of cardiovascular disease [115].

**Table 2:** Overview of sensor technologies for monitoring metabolites in body fluids.

Analyte	Sensor Construction	Body Fluid	Method of Detection	Limit of Detection	Reference
Glucose	Wearable: microneedle (MN)-based assays	interstitial fluid (ISF)	electrochemical	7.44 mM	[100]
Glucose	Wearable platform	sweat	electrochemical	-	[116]
Glucose	Thin-film holographic sensor	blood serum	optical	3 mM	[99]
Glucose	Wearable sensor array	sweat	electrochemical	2.35 nA/μM (sensitivity)	[117]
Lactate	Wearable: microneedle (MN)-based array	interstitial fluid (ISF)	electrochemical	4.43 mM	[100]
Lactate	Eyeglasses-based wireless sensor platform	sweat	electrochemical	0.39 mM	[101]
Lactate	Microneedle Array	blood	electrochemical	-	[118]
Lactate	Microneedle sensor patch	interstitial fluid (ISF)	electrochemical	0.25 mM	[102]
Lactate	Wearable sensing interface	sweat	electrochemical	1 mM	[119]
Lactate	Wearable sensor array	sweat	electrochemical	220 nA/mM (sensitivity)	[117]
Lactate	Temporary-transfer tattoo	sweat	electrochemical	1 mM	[103]
Uric acid	Microfluidic-based plasmonic microneedle	interstitial fluid (ISF)	Surface-Enhanced Raman Spectroscopy	0.51 µM	[109]
Uric acid	Screen-printed electrode modified with gold nanoparticles (SPE-AuNps)	saliva	electrochemical	11.91 μM	[110]
Uric acid	green synthesized silver nanoparticles (Ag NPs)	blood	colorimetric	0.004 μM	[120]
Uric acid	Laser-engraved wearable sensor	sweat	electrochemical	0.74 μM	[111]
Creatinine	Self-powered piezoelectric biosensor	sweat	piezoelectric	1 × 10^−5^ mM	[112]
Cholesterol	Skin-worn microneedle sensor	sweat	electrochemical	0.5 μM	[114]
Cholesterol	Smart contact lens	tears	electrochemical	9.91 μm	[29]

### 5.2. Electrolytes

Electrolytes, including sodium (Na⁺), potassium (K⁺), calcium (Ca^2^⁺), chloride (Cl⁻), and bicarbonate (HCO_3_⁻), are essential ions that regulate the fluid balance, nerve conduction, muscle function, and acid–base homeostasis in the human body [121]. Imbalances in electrolyte concentrations are indicative of various pathological conditions, such as dehydration, kidney dysfunction, cardiac arrhythmia, and endocrine disorders. The quantity of hydrogen ions also plays a crucial role in human body homeostasis. The pH of body fluids is a critical physiological parameter that reflects the acid–base balance, which is essential for normal cellular function and metabolic processes. Different body fluids exhibit characteristic pH ranges: blood maintains a tightly regulated pH of approximately 7.35–7.45, while saliva, sweat, urine, and interstitial fluid show broader variability depending on the individual’s physiological and pathological states. Deviations from normal pH values can indicate underlying health conditions—such as acidosis or alkalosis in when deviations occur in the blood, or urinary tract infections and renal dysfunction when deviations occur in urine. PH and electrolyte levels are usually monitored with wearable sensors the analyze sweat and interstitial fluid. Amongst the work that has been presented so far in the literature, the wearable sensors that we should emphasize include eyeglasses-based sensors [98], tattoos [122], and stretchable, flexible electrochemical platforms [123,124]. Such innovations are particularly valuable in clinical settings, sports medicine, and remote patient monitoring, and contribute to timely diagnosis and individualized care [125,126,127]. Exemplary sensors for electrolyte level and pH monitoring are presented in Table 3.

**Table 3:** Overview of sensor technologies for monitoring electrolytes in body fluids.

Analyte	Sensor Construction	Body Fluid	Method of Detection	Limit of Detection	Reference
Potassium	Eyeglasses-based wireless sensor platform	sweat	electrochemical	10^−3.9^ M	[101]
Potassium	Wearable sensor array	sweat	electrochemical	1 mM	[117]
Sodium	Wearable sensor array	sweat	electrochemical	10 mM	[117]
Sodium	Epidermal tattoo	sweat	electrochemical	-	[122]
Sodium	Wearable platform	sweat	electrochemical	-	[18]
Sodium	Fluorescent dermal tattoo	interstitial fluid	optical	100 mmol/L	[128]
Potassium	Fluorescent dermal tattoo	interstitial fluid	optical	2 mmol/L	[128]
Hydrogen	Fluorescent dermal tattoo	interstitial fluid	optical	6.6 (pH)	[128]
Hydrogen	Wearable Electrochemical Platform	sweat, urine, tears	electrochemical	-	[124]
Calcium	Wearable Electrochemical Platform	sweat, urine, tears	electrochemical	-	[124]
Hydrogen	Potentiometric Nanosensor	interstitial fluid	electrochemical	6.0 (pH)	[129]
Hydrogen	Graphene Field-Effect Transistor (GFET)	electrolyte artificial solution	FET	5.3 (pH)	[130]
Hydrogen	Stretchable wireless system	sweat	electrochemical	5.0 (pH)	[123]

### 5.3. Proteins and Peptides

Proteins and peptides that are present in body fluids serve as critical biomarkers for a wide range of physiological and pathological processes, including inflammation, infection, cancer, and neurodegenerative diseases. These biomolecules include enzymes, hormones, cytokines, and antibodies, which are typically found in the blood, but are also found in saliva, urine, sweat, tears, and cerebrospinal fluid. Monitoring their concentration provides essential insights into disease progression, immune responses, and the efficacy of therapeutic approaches [131]. In this group, we can include C-reactive protein (CRP), prostate-specific antigen (PSA), troponin—a cardiac injury marker, albumin—for kidney and liver function, and cytokines (e.g., TNF-α)—for monitoring immune responses and enzymes (e.g., amylase, ALT, AST). In Table 4, exemplary microsensors and nanosensors for CRP and PSA analysis are reviewed and compared.

C-reactive protein (CRP) is an acute-phase protein that is produced by the liver in response to inflammation and is widely used as a biomarker for systemic inflammation and infection. Elevated CRP levels are associated with conditions such as bacterial infections, autoimmune diseases, and cardiovascular disorders, making CRP a valuable tool in clinical diagnostics and disease monitoring. CRP is typically measured in the blood serum, but recent research has explored its detection in sweat as a less invasive alternative [132,133]. The utilization of microfluidic sensors allowed for the limit of detection of CRP to be lowered to 1 μg/mL [134].

Prostate-specific antigen (PSA) is a glycoprotein enzyme that is primarily produced by the prostate gland, and its elevated levels in blood serum are commonly used as a biomarker for prostate cancer screening, as well as for monitoring benign prostatic hyperplasia (BPH) and prostatitis [135,136]. The implementation of the electrochemical detection of PSA in blood serum made it possible to obtain a limit of detection of 0.035 pg/mL [137], while the implementation of an optical detector allowed a limit as low as 0.145 fg/mL [138].

**Table 4:** Overview of sensor technologies for monitoring proteins in body fluids.

Analyte	Sensor Construction	Body Fluid	Method of Detection	Limit of Detection	Reference
CRP	Microfluidic wireless patch	sweat	electrochemical	-	[139]
CRP	Microfluidic Chip	blood	optical	1 μg/mL	[134]
PSA	PEG/PEDOT nanocomposite—based biosensors	serum	electrochemical	0.035 pg/mL	[137]
PSA	optical biosensor	blood	optical	0.145 fg/mL	[138]

### 5.4. Hormones

Hormones are critical signaling molecules that regulate a wide array of physiological functions, including metabolism, growth, reproduction, and stress responses. Abnormal hormone levels can be indicative of disorders such as diabetes, thyroid dysfunction, adrenal insufficiency, and infertility [140]. Although they are traditionally measured in the blood serum, several hormones, such as cortisol, estradiol, testosterone, and insulin, are also detectable in other body fluids like saliva, urine, and sweat, which offers non-invasive and real-time monitoring opportunities. This group of substances includes cortisol—a stress biomarker, insulin—for diabetes care, testosterone and estrogen—which indicate reproductive and endocrine function, and thyroid hormones (T3, T4, TSH).

Cortisol is a steroid hormone that is produced by the adrenal glands in response to stress and low blood-glucose concentrations. Often referred to as the “stress hormone”, it plays a critical role in various physiological processes, including metabolism regulation, immune response modulation, and maintaining homeostasis. Abnormal levels of cortisol are associated with several health conditions such as Cushing’s syndrome, Addison’s disease, and chronic stress-related disorders [141]. Thanks to the implementation of modern microsensors and nanosensors, cortisol monitoring has been conducted not only by invasive blood testing, but also in saliva and sweat through the use of wearable platforms. Various design solutions for sensors that enable cortisol monitoring have been summarized in Table 5.

**Table 5:** Overview of sensor technologies for monitoring hormones in body fluids.

Analyte	Sensor Construction	Body Fluid	Method of Detection	Limit of Detection	Reference
Cortisol	Graphene flexible sensor array	saliva, sweat	electrochemical	0.08 ng/mL	[142]
Cortisol	MIP stretchable sensor	sweat	electrochemical	0.2 × 10^−9^ M	[143]
Cortisol	Wearable microcapillary channel array	sweat	electrochemical	-	[144]
Cortisol	Field-Effect Transistor sensor	sweat	FET	1 ng/mL	[145]
Interleukin-6	Room Temperature Ionic Liquids (RTILs)—based sensor	sweat	optical	0.2 pg/mL	[146]

### 5.5. Nucleic Acids

Nucleic acids, including DNA and RNA, are the genetic materials of both hosts and pathogens, and serve as molecular biomarkers for detecting infections and genetic disorders. Monitoring pathogen-specific nucleic acids—such as viral RNA (e.g., SARS-CoV-2) or bacterial DNA (e.g., Mycobacterium tuberculosis)—enables highly specific and early disease detection [147]. In recent years, due to the outbreak of COVID-19, numerous biosensors for SARS-CoV-2 virus detection were developed. Sensors that are designed for this purpose utilize various methods of detection such as electrochemical [148], optical [149], or field-effect transistor-based [150] methods. Representative SARS-CoV-2-selective sensors are reviewed in Table 6.

**Table 6:** Overview of sensor technologies for monitoring SARS-CoV-2 in body fluids.

Analyte	Sensor Construction	Body Fluid	Method of Detection	Limit of Detection	Reference
SARS-CoV-2	Electrochemical microsensor	blood serum	electrochemical	0.01 ng/mL	[148]
SARS-CoV-2	Microcavity-based Optical Fiber sensor	blood serum	optical	Single ng/mL	[149]
SARS-CoV-2	nanobody-based photonic nanosensor	blood serum	optical	598 FFU/mL	[151]
SARS-CoV-2	Field-Fffect Transistor (FET)-based biosensing device	blood serum	FET	2.42 × 10^2^ copies/mL	[150]

### 5.6. Drugs and Therapeutic Monitoring

Therapeutic drug monitoring (TDM) through the analysis of body fluids plays a crucial role in optimizing pharmacological treatments, especially for drugs with narrow therapeutic windows or variable pharmacokinetics [152,153,154]. Body fluids such as blood, saliva, urine, and interstitial fluid are commonly used to measure drug concentrations and assess patients’ compliance, drug metabolism, and toxicity risk. For instance, monitoring antiepileptic drugs, antibiotics, immunosuppressants, or chemotherapeutics in the blood allows for dose adjustments that are tailored to individual responses. With the implementation of the electrochemical method of detection, various wearable sensors were designed for the monitoring of levodopa and caffeine. Table 7 provides a comparison of wearable sensors for the monitoring of both drugs.

**Table 7:** Overview of sensor technologies for monitoring drugs in body fluids.

Analyte	Sensor Construction	Body Fluid	Method of Detection	Limit of Detection	Reference
Levodopa	Wearable sweat band on a nanodendritic platform	sweat	electrochemical	1.25 μM	[155]
Levodopa	Wearable patch	sweat	electrochemical	-	[156]
Levodopa	Wearable Electrochemical Microneedle Sensor	interstitial fluid (ISF)	electrochemical	0.5 μM	[157]
Caffeine	Wearable sensor	sweat	electrochemical	3 × 10^−6^ M	[158]

## 6. Emerging Technologies and Trends

Advancements in nanosensors and microsensors are increasingly being shaped by artificial intelligence (AI), the internet of things (IoT), flexible electronics, and non-invasive sensing methods. These innovations are powering the next generation of real-time, smart, and wearable biosensors, making healthcare monitoring more accessible and efficient.

### 6.1. Artificial Intelligence and Machine Learning for Sensor Data Processing

AI and machine learning have significantly improved the analysis and accuracy of sensor data, as well as real-time decision-making. Traditional biosensors generate vast amounts of raw data, which require advanced algorithms for meaningful insights to be extracted. Machine learning models can detect patterns in biosensor data, helping in the early detection of biomarkers [159]. AI-driven self-calibrating biosensors can dynamically adjust for environmental variations, improving the long-term reliability of sensors for this purpose [160]. Moreover, by integrating biosensor data with AI, predictive analytics can be used to provide personalized insights into disease progression and make real-time health recommendations [161,162].

While AI has been widely adopted for analyzing large volumes of complex biosensor data, for example to identify patterns, predict disease onset, or customize therapeutic recommendations, recent advances show that AI can also play an active role in signal acquisition, preprocessing, and even sensor calibration and control. For instance, AI algorithms, particularly neural networks and support vector machines, are increasingly being embedded in edge computing architectures to drive real-time sensor responses, adjust signal amplification, filter noise, and even detect anomalous signals at the hardware level. In smart lab-on-a-chip systems, AI can automate fluidic operations, control actuation, and guide adaptive sensing through feedback loops. Similarly, in closed-loop wearable devices, AI can modulate stimulation (e.g., insulin delivery or electrical stimulation) based on continuously sensed biochemical changes [163].

Therefore, AI is not only a data interpretation tool but a multifunctional enabler within sensor platforms—contributing to signal detection, enhancement, decision-making, and actuation. As microsensor systems evolve into autonomous diagnostic tools, AI will increasingly become central to both upstream (signal-level) and downstream (interpretation-level) operations.

### 6.2. Internet of Things (IoT) and Wireless Sensing

The integration of nanosensors and microsensors with the internet of things (IoT) is enabling remote and continuous health monitoring. Wireless sensing platforms connect biosensors to mobile devices, cloud storage, and medical databases, facilitating real-time healthcare interventions. One of the key advancements in this field is the fabrication of wearable IoT-enabled sensors [164]. Devices such as smartwatches and skin patches can be used to continuously transmit a wearer’s heart rate, glucose levels, and hydration status to cloud-based systems. Another example of the incorporation of innovative technologies into body fluid monitoring is IoT-powered smart lenses that can measure the glucose and electrolyte levels in tears, offering a non-invasive alternative for diabetes monitoring [28].

### 6.3. Flexible and Stretchable Sensors

Traditional biosensors often use rigid materials, which limits their wearability and comfort. Flexible and stretchable sensors designed using nanomaterials, liquid metals, and conductive polymers offer seamless integration with human skin and tissues. An example of the use of nanomaterials is the use of ultra-thin, flexible graphene oxide nanosheets to enable highly sensitive lactate monitoring in sweat [165]. Another implemented technology is self-healing electronic skins. Stretchable electronic tattoos and bio-patches mimic human skin and self-repair minor damage, enhancing the lifespan of sensors [166]. To permit real-time monitoring, flexible microneedles for painless monitoring were invented. Microfluidic microneedles non-invasively extract interstitial fluid for real-time biomarker detection [167,168].

## 7. Challenges and Future Perspectives

While nanosensors and microsensors have revolutionized body fluid monitoring, several challenges remain that must be addressed for them to be widely adopted in clinical and personal healthcare settings. This section discusses key challenges and suggests future research directions to enhance the performance of these sensors and their ease of use.

### 7.1. Biocompatibility, Stability and Reversibility Issues

One of the primary challenges in developing nanosensors and microsensors is ensuring their biocompatibility and long-term stability in biological environments. Since these sensors often interact with body fluids, they must be non-toxic and should not trigger immune responses. The first challenge at the stage of the design of sensors is the selection of appropriate materials. Traditional sensor materials may degrade over time or elicit adverse reactions. The development of biocompatible coatings, such as hydrogels, polydopamine layers, and polyethylene glycol (PEG) modifications, could improve the stability of sensors [169]. One of the greatest challenges in developing wearable biosensors lies in ensuring their long-term functionality. This issue is particularly relevant to implantable sensors. The long-term implantation of sensors poses risks such as biofouling, where proteins and other biomolecules accumulate on the sensor surface, impairing its performance [170]. Strategies such as self-cleaning surfaces and anti-fouling nanocoatings are being explored [171,172].

Another challenge in the practical application of microsensors and nanosensors is the reversibility of the sensing process. Many sensors rely on immobilized probes (e.g., aptamers, antibodies, or molecularly imprinted polymers) that bind selectively to target analytes. In some cases, this interaction is so strong that it results in poor reversibility or requires harsh chemical or thermal treatments to regenerate the sensing surface. This can limit the reusability of sensors, particularly in continuous or long-term monitoring scenarios. Materials that exhibit reversible binding through non-covalent interactions, such as supramolecular systems or dynamic polymer networks, are being explored to overcome this limitation [173,174,175].

### 7.2. Calibration and Interferences

To provide accurate and reliable readings, nanosensors and microsensors require precise calibration and high sensitivity. However, the changing nature of body fluids can cause variability that impacts the performance of sensors. First, there is a challenge related to interference coming from, for instance, other biomolecules. Body fluids contain a complex matrix of biomolecules, ions, and proteins that can interfere with sensor readings. Developing highly selective nanomaterials, such as molecularly imprinted polymers (MIPs), can enhance the specificity of sensors [62,63,64,65,66,67]. Over time, sensor readings may exhibit drift due to environmental changes and thus require frequent recalibration. Researchers are exploring self-calibrating biosensors that utilize machine learning to correct drift and environmental fluctuations [160]. Another major hurdle in the case of microsensors and nanosensors is miniaturization. While miniaturization improves the wearability of sensors, it can sometimes reduce their sensitivity. Advanced graphene-based transistors are being investigated to enhance their signal detection at the nano scale [176,177,178].

### 7.3. Real-Time Data Processing and Interpretation

With the increasing use of wearable and implantable sensors, real-time data processing is becoming a critical challenge. Continuous monitoring generates vast amounts of health data. Efficient data compression algorithms and cloud-based processing systems are needed to handle this issue [179]. AI and machine learning are being integrated into nanosensor platforms to identify patterns and detect anomalies in biosensor data. These models can help predict disease progression and personalize treatment plans [180]. Moreover, many wearable sensors rely on battery power; hence, there is a need for the development of low-energy signal processing units or self-powered nanosensors [9,43,112].

### 7.4. Regulatory and Ethical Considerations

The transition of nanosensors and microsensors from research to commercial use is subject to regulatory approvals and ethical considerations. There is a lack of standardized testing protocols for evaluating the accuracy, safety, and longevity of nanosensors. Regulatory bodies such as the FDA (Food and Drug Administration) are working to establish guidelines for these technologies. Wearable and implantable sensors collect sensitive health data, which raises concerns about cybersecurity [181,182]. There is also a challenge regarding ethical issues in the integration of AI with sensors. AI-driven nanosensors must be transparent in their decision-making processes [183].

## 8. Conclusions

Nanosensors and microsensors have revolutionized real-time body-fluid monitoring, offering non-invasive, highly sensitive, and personalized diagnostic solutions. This review has highlighted several key advancements and challenges associated with these types of sensors, including their importance in healthcare, technological advancements, their application in point-of-care treatment, and challenges and limitations.

Nanosensors and microsensors provide real-time insights into physiological states by detecting critical biomarkers in fluids such as blood, sweat, saliva, urine, and interstitial fluids. They enable early disease detection and continuous health monitoring. The use of nanomaterials (e.g., graphene, gold nanoparticles, quantum dots), microfluidics, and AI integration has significantly improved the performance of these sensors, enhancing their sensitivity, selectivity, and stability. These sensors have demonstrated promising applications in diabetes management (continuous glucose monitoring), cardiovascular disease detection, electrolyte and hydration monitoring, and infectious disease diagnosis.

Despite their potential, numerous challenges must be addressed before they achieve widespread clinical adoption. Biocompatibility, calibration, real-time data processing, regulatory approval, and ethical concerns remain key areas of focus. However, advancements in nanomaterials, AI-driven data analytics, and self-powered sensor systems are rapidly overcoming these barriers. The integration of AI, the IoT, and machine learning with microsensors and nanosensors will enhance real-time data interpretation, enabling more effective predictive diagnostics and automated health recommendations. Due to the implementation of flexible and wearable materials, the development of skin-integrated, stretchable, and biocompatible sensors will facilitate continuous health tracking in a comfortable way.

With ongoing research and innovation, nanosensors and microsensors will play a crucial role in shifting from hospital-based diagnostics to at-home monitoring. Early detection helps prevent disease progression and reduces healthcare costs. In conclusion, while challenges remain, the continuous evolution of nanosensor and microsensor technologies will redefine healthcare diagnostics, enhance patient outcomes, and pave the way for a new era of digital and personalized medicine.

## Figures and Tables

**Table 1:** Comparative summary of key transduction mechanisms used in nanosensors and microsensors for body fluid monitoring.

Transduction Type	Sensitivity	Integration (Wearable)	Integration (Ingestible)	Cost	Real-Time Use	Miniaturization
Electrochemical	High	Excellent	Moderate	Low	Yes	Excellent
Optical	High	Moderate	Challenging	Medium	Yes	Good
Piezoelectric	Moderate	Moderate	Low	Medium	Limited	Moderate
FET-based	Very High	Emerging	Research-stage	High	Yes	Excellent

## Abbreviations

The following abbreviations are used in this manuscript:

MEMS	microelectromechanical systems
LOC	lab-on-a-chip
LOP	lab-on-a-patch
SoC	systems-on-chip
SiP	system-in-package
MOEMS	micro-optoelectromechanical systems
IoT	internet of things
GMC	continuous glucose monitoring
PSA	prostate-specific antigen
ISF	interstitial fluid
FET	field-effect transistor
CQDs	carbon quantum dots
QDs	quantum dots
SiNW	silicon nanowires
NFC	near-field communication
CNTs	carbon nanotubes
GO	graphene oxide
DNA	deoxyribonucleic acid
SERS	surface-enhanced raman spectroscopy
PANI	polyaniline
PPy	polypyrrole
PT	polythiophene
PDMS	polydimethylsiloxane
PEDOT	poly(3,4-ethylenedioxythiophene
MIPs	molecularly imprinted polymers
µTAS	miniaturized total analysis system
POC	point-of-care
AuNR	gold nanorods
GFR	glomerular filtration rate
CKD	chronic kidney disease
MN	microneedle
NPs	nanoparticles
SPE	Screen-printed electrode
CRP	C-reactive protein
IL-6	interleukin-6
RNA	ribonucleic acid
SARS-CoV-2	severe acute respiratory syndrome coronavirus 2
TDM	therapeutic drug monitoring
AI	artificial intelligence
PEG	polyethylene glycol
FDA	Food and Drug Administration
